# Nuclear and Cellular Abnormalities of Erythrocytes in Response to Thermal Stress in Common Carp *Cyprinus carpio*

**DOI:** 10.3389/fphys.2020.00543

**Published:** 2020-06-05

**Authors:** Md. Shahjahan, Most. Sabia Khatun, Mim Mostarin Mun, S. M. Majharul Islam, Md. Helal Uddin, Muhammad Badruzzaman, Saleha Khan

**Affiliations:** ^1^Laboratory of Fish Ecophysiology, Department of Fisheries Management, Bangladesh Agricultural University, Mymensingh, Bangladesh; ^2^Department of Biochemistry and Molecular Biology, Bangabandhu Sheikh Mujibur Rahman Agricultural University, Gazipur, Bangladesh

**Keywords:** common carp, temperature, blood glucose, hemoglobin, erythrocytes, leukocytes

## Abstract

As a consequence of global warming, increase of water temperature is likely to alter physiological functions of fish. Hence, we examined the effects of high temperature on blood glucose, hematological parameters [hemoglobin (Hb), red blood cell (RBC), and white blood cell (WBC)], and nuclear and cellular structure of blood cells of common carp (*Cyprinus carpio*) after exposure to three temperature regimes (27, 31, and 35°C) for 14 days. Fish were sacrificed on 3, 7, and 14 days of exposure. The blood glucose level increased significantly in the fish exposed to 35°C compared to 27 and 31°C. The Hb and RBC contents decreased but WBC increased significantly in the blood of fish exposed to 35°C compared to 27 and 31°C at 7 and 14 days of exposure. Consequently, the frequencies of erythroblasts (Ebs), erythrocytic nuclear abnormalities (ENA), and erythrocytic cellular abnormalities (ECA) were found to be increased in the blood of fish exposed to 35°C compared to 27 and 31°C. There was a significant increase in neutrophils and decrease in lymphocytes in the highest temperature (35°C). With increasing temperature, dissolved oxygen (DO) decreased but free CO_2_ increased significantly during the study period. The present study demonstrated that common carp are better adapted to 27 and 31°C environmental temperatures, while the higher temperature 35°C is likely stressful to this fish species.

## Introduction

The common carp (*Cyprinus carpio*) is a native fish species in temperate regions of Asia, especially in China (Gunther, 1868), Turkestan (Jenkins, 1961), and Japan (Okada, 1960). This fish species has been acclimatized to various environments and habitats. Therefore, it has global distribution including tropical regions. In Bangladesh, the common carp was introduced in mid-fifties and well established in the waters of valley areas, especially in the lentic habitats. Currently, this fish species is more abundant than nearly all indigenous fish species (Hasan et al., 2007). Its high growth rate and productive breeding in confined water make highly edible and ideal cultivable species in the world. It is the third most widely cultured freshwater fish species in the world (FAO, 2018). During the year 2018, the contribution of common carp to the global aquaculture production was nearly 9.0% (FAO, 2018). Similarly, the contribution of common carp as a single species was 3.2% during the year 2016–2017 in Bangladesh (Fisheries Resources Survey System, 2017). The ecological spectrum of carp is broad. Best growth is obtained when water temperature ranges between 23 and 30°C (Sapkale et al., 2011; Oyugi et al., 2012).

The distribution, survivability, and different physiological activities of fishes and other heterotherms are greatly influenced by various environmental factors. Temperature plays a critical role in the growth and reproduction of fish (Cheng et al., 2013; Shahjahan et al., 2013, 2017). Nonetheless, climate caused increase of water temperature is great concern in the recent decades and is expected to affect physiology of fishes (Chatterjee et al., 2004; Pörtner and Farrell, 2008; Verhille et al., 2016; Fu et al., 2018). This is especially alarming for fishes of tropical regions due to the higher route of changes in these environments (Deutsch et al., 2008). Temperature may increase to a level that could be detrimental for growth and different physiological process in aquatic organisms (Fu et al., 2018), as environmental temperature regulates the overall performances of poikilothermic animals like fish (Angilletta, 2009). For every 10°C increase of temperature, roughly the rates of biochemical processes double in fish (Boyd and Tucker, 1998). Therefore, it is justified to study how temperature changes, especially high temperature affects the physiology of fish. The impacts of temperature changes on fish could be anticipated by study of their physiological activities (Somero, 2010). It has been reported that physiological processes are greatly affected when temperature exceeded the level of tolerance (Chatterjee et al., 2004).

Different blood parameters are commonly measured to assess the physiological conditions of fish after exposure to different environmental stressors, including temperature (Affonso et al., 2002; Salam et al., 2015; Sharmin et al., 2015; Sadiqul et al., 2016; Shahjahan et al., 2019). Hemoglobin (Hb) contents, the number and size of erythrocytes in the blood vary with changes of temperature (Andersen et al., 1985; Ytrestoyl et al., 2001). It has been reported that Hb content increased in a situation of decreased dissolved oxygen (DO) and increased metabolic activity due to increases of water temperature in the Atlantic cod (Brix et al., 2004). Moreover, morphological alterations (cellular and nuclear) of erythrocytes and formation of differential leukocytes count are also used as biomarkers to assess the stress caused by any environmental factors (Ghaffar et al., 2015; Sadiqul et al., 2016; Shahjahan et al., 2018). In the recent past, micronucleus formation in the blood cells is used to assess the stress caused by environmental pollutants (Sadiqul et al., 2016; Islam S. et al., 2019; Shahjahan et al., 2019).

Although several studies were conducted to understand the impacts of thermal stress to physiological responses of fish (Bevelhimer and Bennett, 2000; Fu et al., 2018; Shahjahan et al., 2018; Islam M. et al., 2019), there is no report on the effects of high temperature on any physiological activities in the common carp which is a commercial aquaculture fish species throughout the world. Therefore, the aim of this experiment was to evaluate the impacts of high water temperature on blood glucose level, hematological parameters, morphology of erythrocytes, and formation of different leukocytes in the common carp (*C. carpio*).

## Materials and Methods

### Experimental Fish

Sexually mature active, healthy, and disease free common carp (*C. carpio*) was procured from a local fish farm. The average weight and length of the fish was 57.64 ± 0.78 g and 16.55 ± 0.60 cm, respectively. The fish was acclimatized in the laboratory for 21 days before start of experiment at 27°C. During acclimatization, the fish was fed twice a day up to satiation with commercial feed (Quality Fish Feed Ltd., Bangladesh).

### Experimental Design

The fish were exposed to three different temperatures, including 27 (control), 31 and 35°C in three replications. Nine glass aquaria (75 cm × 45 cm × 45 cm), each filled with 100 L of tap water were used to conduct this experiment. Ten fish were stocked in each aquarium. Filtration-cum aeration device was set in the aquarium for self-cleaning and aeration during the experimental periods. Temperature was gradually increased 1°C/day from acclimation temperature 27°C to the desired temperatures (31 and 35°C) and maintained using thermostat (REI-SEA, Japan) for 14 days. Stocked fish were fed with commercial pellet feed twice a day up to satiation. The animal welfare and ethical committee of Bangladesh Agricultural University, Mymensingh, approved the experimental procedure used in this study.

### Blood Sampling

At days 3, 7, and 14 of exposure to three temperature conditions, six fish (*n* = 6) were sacrificed from each temperature regime. Fish were anesthetized by clove oil (5 mg/L) immediately after collection from aquaria. Blood from each fish was sampled from the caudal peduncle and stored in an Eppendorf tube having anticoagulant (20 mM EDTA) until further use for counting of red blood cell (RBC) and white blood cell (WBC).

### Measurement of Blood Glucose Level

Immediately after collection of blood samples, glucose (mg/dL) content was assessed by a digital EasyMate^®^ GHb (Model-ET, 232) monitoring system using glucose strip.

### Measurement of Hematological Parameters

Hemoglobin (g/dL) content was estimated using a SAHLI’s hemometer following standard protocol. In brief, 90 μL 0.1 N HCl and 10 μL blood was taken in an Eppendorf tube using micropipette and mixed properly by shaking. The mixture was transferred to the tube of the hemometer. Distilled water was added in drops until the color was adjusted with the colorimeter of the hemometer. The reading was taken up to the level of the mixture showed on the body of the tube. The numbers of RBC and WBC were counted using a Neubauer hemocytometer under a light microscope following standard protocol.

### Analysis of Morphological Changes of Erythrocytes and Differential Leukocyte Count

The procedures for analysis of morphological changes of erythrocytes and differential leukocytes count were described in detail by Jahan et al. (2019) and Shahjahan et al. (2019). In brief, blood was smeared on glass slides immediately after collection from the fish. The slides were stained by 5% Giemsa stain after fixed with methanol for 10 min. Frequencies of different morphological changes of erythrocytes, including erythroblasts (Ebs) (pro, basophilic, polychromatophilic, and orthochromatic Eb), erythrocytic nuclear abnormalities (ENA), erythrocytic cellular abnormalities (ECA), and differential leukocytes were observed in the smeared blood under an electronic microscope (MCX100, Micros Austria).

Erythrocytic nuclear abnormalities were classified according to Carrasco et al. (1990). Briefly, different types of ENA were described as micronucleus: circular chromatin bodies showed the staining shape alike to the central nucleus; nuclear bud: nuclei with bud-like evaginations; binucleated: cell with two nuclei; karyopyknosis: cells with condensation and clumping of the chromatin materials in the periphery of the nuclei along with irregular nuclear membranes; and notched nucleus that did not contain nuclear material. ECA was classified as twin: two cells joined by the cell surface; tear−drop shape: an erythrocyte deformed and tugged to a nipple shape at one end; elongated: having notably more unusual length than width, being longer and slender shaped; fusion: the attaching (joining) of more than two cells to form a heavier cell mass and volume; echinocytic: having serrated boundaries over the entire cell surface while uniform in shape; and spindle, being more or less circular within the center with two pointed closes.

Frequencies of different types of leukocyte, including monocytes, neutrophils, lymphocytes, and eosinophils were counted from the prepared smeared slides.

### Water Quality Parameters

Dissolved oxygen, pH, free CO_2_, and total alkalinity were recorded throughout the experiment conducted period. Digital DO meter and pH meter were used to monitor these two water quality parameters, respectively. Titrimetric method was applied to assess the free CO_2_ and total alkalinity of water using different specific indicators and titrants.

### Statistical Analysis

Values of all the measured variables are articulated as means ± SD. Before statistical analyses, the normality and homogeneity of variance were tested for all groups of data. Statistical significant difference among three temperatures and days of exposure were tested by two-way analysis of variance (ANOVA). All statistical analyses were performed using PASW statistics 18.0 software (IBM, Chicago, IL, United States) setting significance at *p* < 0.05.

## Results

### Changes of Blood Glucose Level

**FIGURE 1:**
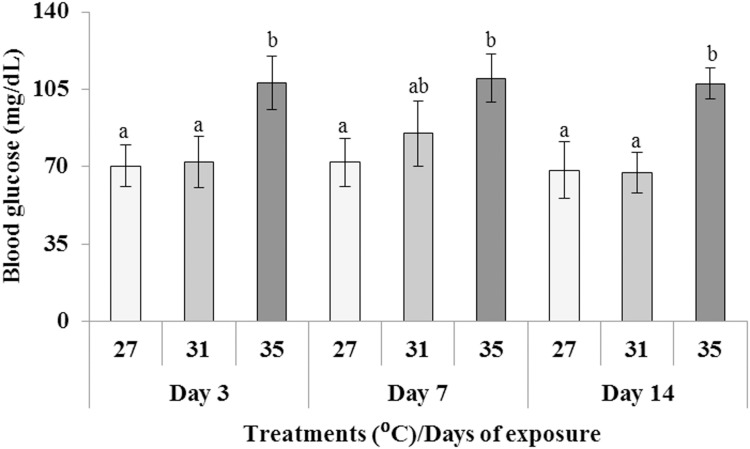
Changes in blood glucose level (mg/dL) after exposure to three different temperature conditions. Values with different alphabetical superscripts are significantly (*p* < 0.05) different. All values expressed as mean ± SD (*n* = 6).

Fish blood glucose levels were assessed after 3, 7, and 14 days of exposure at three different temperatures (27, 31, and 35°C). The blood glucose level (mg/dL) of fish exposed to 35°C elevated significantly (*p* < 0.05) compared to those in 27 and 31°C during all the sampling days (Figure 1).

### Changes in Hematological Parameters

The percentage of Hb and amount of RBC and WBC in fish blood was calculated after 3, 7, and 14 days of exposure to three different temperature levels (27, 31, and 35°C). In days 7 and 14, the values of Hb (g/dL) and number of RBC decreased significantly (*p* < 0.05) in fish treated with 35°C compared to 27 and 31°C, while in day 3, no distinct changes were found among three temperature treated groups (Table 1). On the other hand, WBC number revealed the opposite scenario (Table 1).

**TABLE 1:** Changes in hematological parameters after exposure to three different temperature conditions during the experimental period.

Parameters	Temperature	Sampling days		
		
		3	7	14
Hb (g/dL)	27°C	6.20 ± 0.26	6.37 ± 0.21^a^	6.07 ± 0.12^a^
	31°C	6.27 ± 0.25	6.07 ± 0.23^a^	6.17 ± 0.21^a^
	35°C	5.57 ± 0.31	4.87 ± 0.32^b^	4.23 ± 0.25^b^
RBC	27°C	0.84 ± 0.04	0.92 ± 0.02^a^	0.88 ± 0.09^a^
(×10^6^/mm^3^)	31°C	0.74 ± 0.10	0.85 ± 0.08^ab^	0.94 ± 0.01^a^
	35°C	0.70 ± 0.10	0.63 ± 0.04^b^	0.53 ± 0.07^b^
WBC	27°C	5.9 ± 0.03^a^	5.4 ± 0.11^a^	5.7 ± 0.12^a^
(×10^3^/mm^3^)	31°C	6.1 ± 0.03^a^	5.7 ± 0.13^a^	7.0 ± 0.15^ab^
	35°C	9.7 ± 0.10^b^	8.9 ± 0.12^b^	9.2 ± 0.16^b^

*Values of a single hematological parameter in a column with different alphabetical superscripts are significantly (*p* < 0.05) different. All values expressed as mean ± SD (*n* = 6).*

### Morphological Alterations in Erythrocytes

**FIGURE 2:**
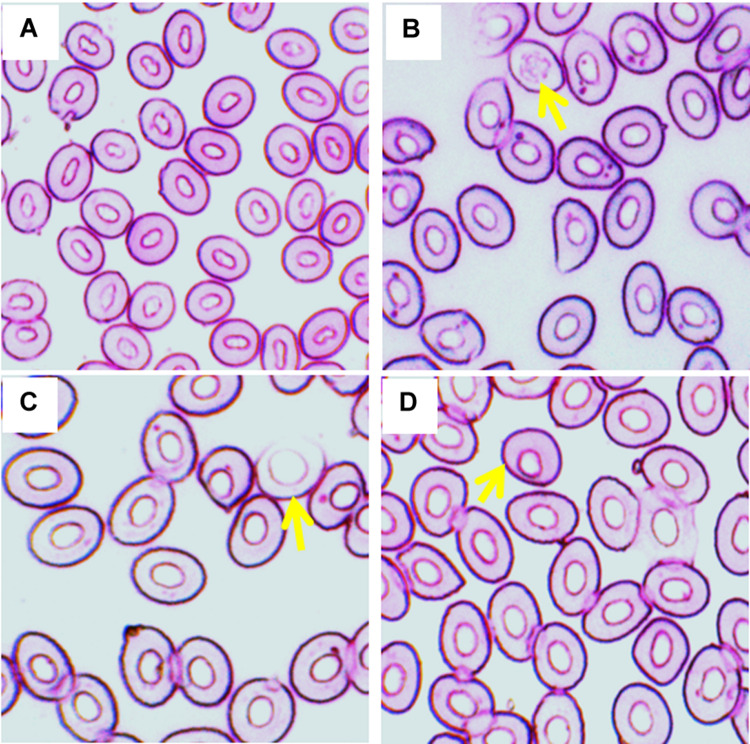
Various erythroblasts (Ebs) in giemsa stained blood smears of fish treated with three different temperature conditions; **(A)** regular cells, **(B)** pro erythroblast, **(C)** basophilic erythroblast, and **(D)** orthochromatophilic erythroblast.

Frequencies of different stages of Ebs, including pro, basophilic, polychromatophilic, and orthochromatic Ebs in the blood of fish reared in different temperature (Figures 2A–D) are shown in Table 2. Statistically significant (*p* < 0.05) increase in Ebs was detected in blood of fish exposed to the highest temperature (35°C). When frequencies of Ebs were compared among different days of exposure, there was a significant higher frequency at day 7 and 14 than those at day 3 in the highest temperature.

**TABLE 2:** Frequencies of erythroblasts (Ebs) after 3, 7, and 14 days of exposure to three different temperature conditions.

Ebs	Temperature	Percentage of Ebs		
		
		Exposure time (day)		
		
		3	7	14
Proery-	27°C	0.29 ± 0.05^a^	0.24 ± 0.01^a^	0.25 ± 0.01^a^
throblast	31°C	0.73 ± 0.09^ab^	0.85 ± 0.05^ab^	0.77 ± 0.03^ab^
	35°C	0.88 ± 0.13^b,1^	1.21 ± 0.11^b,2^	1.02 ± 0.05^b,2^
Basophilic	27°C	0.22 ± 0.03^a^	0.30 ± 0.04^a^	0.19 ± 0.01^a^
erythroblast	31°C	0.57 ± 0.09^ab^	0.70 ± 0.06^ab^	0.46 ± 0.05^a^
	35°C	0.85 ± 0.13^b,1^	1.31 ± 0.12^b,2^	1.05 ± 0.05^b,2^
Polychro-	27°C	0.00 ± 0.00	0.00 ± 0.00	0.00 ± 0.00
matophilic	31°C	0.00 ± 0.00	0.00 ± 0.00	0.00 ± 0.00
erythroblast	35°C	0.00 ± 0.00	0.00 ± 0.00	0.00 ± 0.00
Ortho-	27°C	0.29 ± 0.05^a^	0.32 ± 0.00^a^	0.27 ± 0.03^a^
chromatic	31°C	0.56 ± 0.09^ab^	0.85 ± 0.13^b^	0.53 ± 0.05^ab^
erythroblast	35°C	0.76 ± 0.07^b,1^	1.32 ± 0.17^b,2^	1.30 ± 0.05^b,2^

*Values of a single Ebs in a column with different alphabetical superscripts are significantly (*p* < 0.05) different. Values with different numeric superscripts in a row differ significantly (*p* < 0.05) among days of exposure. All values expressed as mean ± SD. Three slides were prepared from each fish and 2000 cells were scored from each slide and at least three fishes were analyzed from each group.*

**FIGURE 3:**
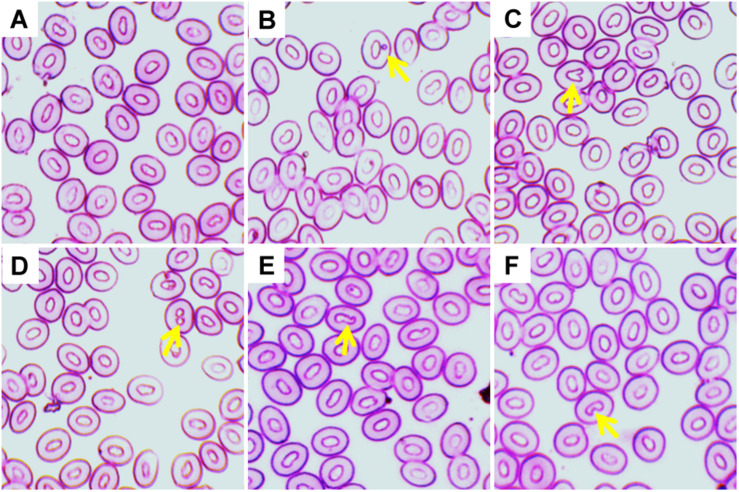
Various erythrocytic nuclear abnormalities (ENA) in Giemsa stained blood smears of common carp treated with three different temperature conditions; **(A)** regular cells, **(B)** micronucleus, **(C)** nuclear bud, **(D)** binucleated, **(E)** karyopyknosis, and **(F)** notched nuclei.

Various ENA, including micronucleus, nuclear bud, binucleated, karyopyknosis, and notched nuclei were observed in Giemsa stained blood smears of common carp treated with three different temperature conditions (Figures 3A–F). The frequencies of different ENA are presented in Table 3. There was a significant (*p* < 0.05) increase in the frequencies of ENA in 35°C treated group compared to 27°C treated group throughout the experimental periods. When frequencies of ENA were compared among different days of exposure, significant lower frequency of binucleated, karyopyknosis, and notched nuclei were found at day 14 than those at day 3 and 7 in the highest temperature.

**TABLE 3:** Frequencies of erythrocytic nuclear abnormalities (ENA) after 3, 7, and 14 days of exposure to three different temperature conditions.

ENA	Temperature	Percentage of ENA		
		
		Exposure time (day)		
		
		3	7	14
Micro-	27°C	0.29 ± 0.05^a^	0.32 ± 0.0^a^	0.25 ± 0.01^a^
nucleus	31°C	0.73 ± 0.09^ab^	0.59 ± 0.05^a^	0.57 ± 0.03^a^
	35°C	0.88 ± 0.13^b^	0.64 ± 0.07^b^	0.52 ± 0.05^b^
Nuclear	27°C	0.22 ± 0.03^a^	0.23 ± 0.01^a^	0.19 ± 0.01^a^
bud	31°C	0.57 ± 0.09^ab^	0.56 ± 0.05^ab^	0.46 ± 0.05^ab^
	35°C	0.85 ± 0.13^b^	0.74 ± 0.05^b^	0.65 ± 0.05^b^
Binuclei	27°C	0.20 ± 0.09^a^	0.26 ± 0.07^a^	0.16 ± 0.05^a^
	31°C	0.57 ± 0.15^ab^	0.40 ± 0.11^ab^	0.34 ± 0.09^a^
	35°C	1.93 ± 0.15^b,1^	1.25 ± 0.09^b,1^	0.98 ± 0.05^b,2^
Karyopy-	27°C	0.29 ± 0.05^a^	0.27 ± 0.03^a^	0.22 ± 0.03^a^
knosis	31°C	0.56 ± 0.09^ab^	0.35 ± 0.05^a^	0.33 ± 0.05^a^
	35°C	0.70 ± 0.07^b,1^	0.71 ± 0.05^b,1^	0.30 ± 0.05^a,2^
Notched	27°C	0.45 ± 0.09^a^	0.51 ± 0.05^a^	0.45 ± 0.07^a^
nuclei	31°C	0.80 ± 0.13^ab^	0.83 ± 0.09^ab^	0.60 ± 0.10^a^
	35°C	1.95 ± 0.15^b,1^	1.64 ± 0.11^b,1^	0.85 ± 0.11^b,2^

*Values of a single ENA in a column with different alphabetical superscripts are significantly (*p* < 0.05) different. Values with different numeric superscripts in a row differ significantly (*p* < 0.05) among days of exposure. All values expressed as mean ± SD. Three slides were prepared from each fish and 2000 cells were scored from each slide and at least three fishes were analyzed from each group.*

**FIGURE 4:**
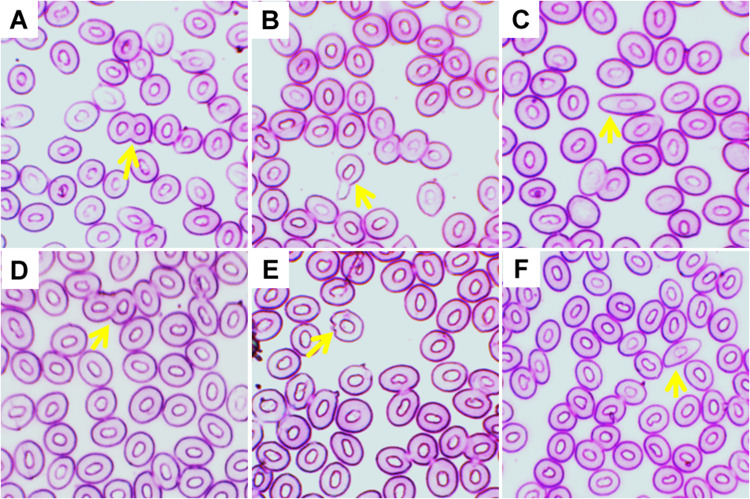
Various erythrocytic cellular abnormalities (ECA) in Giemsa stained blood smears of common carp treated with three different temperature conditions; **(A)** twin, **(B)** tear drop shaped, **(C)** elongated shape, **(D)** fusion, **(E)** echinocytic, and **(F)** spindle.

Different types of ECA, including twin, tear drop shaped, elongated shape, fusion, echinocytic, and spindle were recorded in giemsa stained blood smears of common carp treated with three different temperature conditions (Figures 4A–F). Frequencies of several types of ECA in fish exposed to different temperature conditions are presented in Table 4. Similar to ENA, significant increases (*p* < 0.05) in ECA were found in the blood of fishes exposed to the highest temperature regime (35°C). When incidences of ECA were compared among different days of exposure, there was a significant lower frequency of tear drop shaped and echinocytic cells at day 14 than those at day 3 and 7 in the highest temperature.

**TABLE 4:** Frequencies of erythrocytic cellular abnormalities (ECA) after 3, 7, and 14 days of exposure to three different temperature conditions.

ECA	Temperature	Percentage of ECA		
		
		Exposure time (day)		
		
		3	7	14
Twin	27°C	0.87 ± 0.11^a^	0.99 ± 0.09^a^	0.80 ± 0.07^a^
	31°C	2.51 ± 0.15^ab^	2.08 ± 0.09^ab^	2.10 ± 0.07^ab^
	35°C	3.20 ± 0.18^b,1^	2.89 ± 0.07^b,2^	2.82 ± 0.07^b,2^
Tear-drop	27°C	0.83 ± 0.09^a^	0.77 ± 0.09^a^	0.68 ± 0.09^a^
	31°C	0.87 ± 0.13^ab^	0.82 ± 0.11^a^	0.83 ± 0.10^a^
	35°C	1.53 ± 0.15^b,1^	0.98 ± 0.11^a,2^	0.74 ± 0.09^a,2^
Elongated	27°C	0.30 ± 0.13^a^	0.21 ± 0.09^a^	0.23 ± 0.07^a^
	31°C	0.86 ± 0.09^ab^	0.96 ± 0.05^ab^	0.75 ± 0.05^ab^
	35°C	1.50 ± 0.10^b^	1.35 ± 0.09^b^	0.98 ± 0.05^b^
Fusion	27°C	1.29 ± 0.07^a^	0.87 ± 0.05^a^	0.76 ± 0.09^a^
	31°C	1.52 ± 0.11^ab^	0.80 ± 0.09^a^	0.78 ± 0.07^a^
	35°C	1.89 ± 0.11^b,1^	0.95 ± 0.09^a,2^	0.80 ± 0.10^a,2^
Echino-	27°C	0.20 ± 0.07^a^	0.15 ± 0.05^a^	0.17 ± 0.05^a^
cytic	31°C	0.62 ± 0.13^ab^	0.45 ± 0.09^ab^	0.30 ± 0.09^ab^
	35°C	1.92 ± 0.15^b,1^	1.39 ± 0.09^b,12^	0.80 ± 0.07^b,2^
Spindle	27°C	0.97 ± 0.11^a^	0.89 ± 0.09^a^	0.90 ± 0.07^a^
	31°C	1.51 ± 0.15^ab^	1.08 ± 0.09^ab^	1.10 ± 0.07^ab^
	35°C	2.20 ± 0.18^b^	1.89 ± 0.07^b^	1.82 ± 0.07^b^

*Values of a single ECA in a column with different alphabetical superscripts are significantly (*p* < 0.05) different. Values with different numeric superscripts in a row differ significantly (*p* < 0.05) among days of exposure. All values expressed as mean ± SD. Three slides were prepared from each fish and 2000 cells were scored from each slide and at least three fishes were analyzed from each group.*

### Differential Leukocyte Count

**FIGURE 5:**
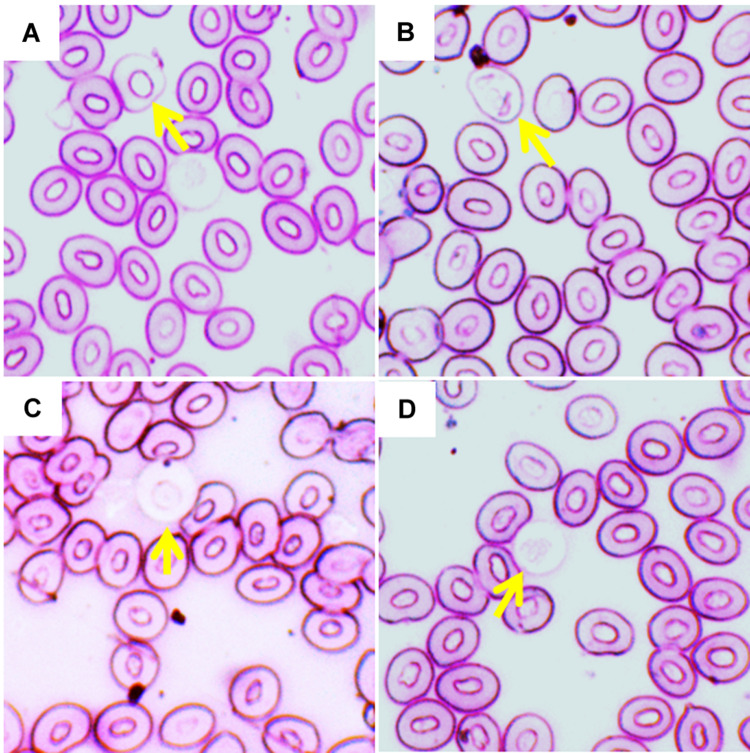
Different leukocyte in Giemsa stained blood smears of common carp treated with three different temperature conditions; **(A)** monocytes, **(B)** neutrophils, **(C)** lymphocytes, and **(D)** eosinophils.

The frequencies of different types of leukocytes, including monocytes, neutrophils, lymphocytes, and eosinophils (Figure 5) to different temperature regimes are shown in Table 5. Interestingly, no basophils were observed in any temperature conditions. The frequencies of occurrences of neutrophils increased but lymphocytes decreased significantly (*p* < 0.05) in fish treated at the highest temperature condition (35°C) compared to the lowest temperature condition (27°C). On the other hand, monocytes and eosinophils showed no variation irrespective of any temperature conditions.

**TABLE 5:** Differential leukocyte count after 3, 7, and 14 days of exposure to three different temperature conditions.

Leucocytes	Temperature	Percentage of leukocytes		
		
		Exposure time (day)		
		
		3	7	14
Monocytes	27°C	6.0 ± 1.0	3.0 ± 1.0	4.0 ± 1.0
	31°C	7.0 ± 1.0	4.0 ± 1.0	4.0 ± 1.0
	35°C	4.0 ± 1.0	5.0 ± 1.0	4.0 ± 1.0
Neutrophil	27°C	35.0 ± 3.0^a^	37.0 ± 3.0^a^	36.0 ± 1.0^a^
	31°C	54.0 ± 3.0^ab^	56.0 ± 3.0^ab^	53.0 ± 3.0^ab^
	35°C	76.0 ± 5.0^b^	74.0 ± 5.0^b^	75.0 ± 5.0^b^
Lymphocytes	27°C	54.0 ± 6.0^b^	57.0 ± 5.0^b^	56.0 ± 1.0^b^
	31°C	33.0 ± 4.0^ab^	35.0 ± 4.0^ab^	38.0 ± 4.0^ab^
	35°C	16.0 ± 2.0^a^	17.0 ± 2.0^a^	18.0 ± 2.0^a^
Eosinophil	27°C	5.0 ± 1.0	3.0 ± 1.0	4.0 ± 1.0
	31°C	6.0 ± 1.0	5.0 ± 1.0	5.0 ± 1.0
	35°C	4.0 ± 1.0	4.0 ± 1.0	3.0 ± 1.0

*Values with different alphabetical superscripts in a column differ significantly (*p* < 0.05) among different temperature in differential leukocytes. All values expressed as mean ± SD.*

### Changes in Water Quality Parameters

The values of DO, free CO_2_, pH, and total alkalinity are shown in Table 6. The DO (mg/L) decreased but free CO_2_ (mg/L) increased significantly (*p* < 0.05) in the highest temperature (35°C). On the other hand, the values of pH and total alkalinity (mg/L) showed no distinct change during the experimental period irrespective of any temperature conditions (Table 6).

**TABLE 6:** Water quality parameters (mean ± SD) during the experimental period.

Parameters	Temperature	Sampling days		
		
		3	7	14
Dissolved	27°C	6.8 ± 0.11^a^	6.9 ± 0.09^a^	6.7 ± 0.09^a^
oxygen	31°C	5.3 ± 0.10^ab^	5.5 ± 0.06^ab^	5.2 ± 0.07^ab^
(mg/L)	35°C	4.2 ± 0.09^b^	4.4 ± 0.05^b^	4.3 ± 0.04^b^
Free	27°C	4.2 ± 0.08^a^	4.0 ± 0.09^a^	3.9 ± 0.08^a^
CO_2_	31°C	5.4 ± 0.10^ab^	5.1 ± 0.10^ab^	4.9 ± 0.09^ab^
(mg/L)	35°C	7.9 ± 0.09^b^	8.0 ± 0.08^b^	8.4 ± 0.10^b^
pH	27°C	8.2 ± 0.08	8.2 ± 0.09	8.3 ± 0.09
	31°C	7.9 ± 0.09	8.1 ± 0.10	8.1 ± 0.10
	35°C	8.0 ± 0.10	8.0 ± 0.08	8.5 ± 0.08
Total	27°C	110 ± 0.09	112 ± 0.08	96 ± 0.09
alkalinity	31°C	104 ± 0.08	96 ± 0.09	107 ± 0.08
(mg/L)	35°C	98 ± 0.10	100 ± 0.10	98 ± 0.10

*Values of a single water quality parameter in a column with different alphabetical superscripts are significantly (*p* < 0.05) different.*

## Discussion

Various physiological functions of teleost are fundamentally affected by the water temperature. The present study demonstrated high temperature induced changes in blood glucose level, hematological parameters, and morphology of blood cells without any mortality of fish. Therefore, the highest temperature (35°C) might be stressful for the common carp. Stress induced the changes of various physiological functions in teleost (Fu et al., 2018; Shahjahan et al., 2018; Ashaf-Ud-Doulah et al., 2019) in agreement with our findings.

Generally, blood glucose are measured as a stress indicator (Pacheco and Santos, 2001; Beyea et al., 2005; Shahjahan et al., 2018). Hence in the highest temperature, increased blood glucose level may be due to transforming glycogen into glucose to satisfy the extra demand for metabolic energy under stressful conditions caused by highest temperature. It has been stated that increased glucose levels are necessary to meet the new energy demands of stressed fish (Winkaler et al., 2007; Ashaf-Ud-Doulah et al., 2019). In addition, thermal stress can have a destructive effect on the functions of major organs including the liver and kidney that impair fish’s homeostasis (Sharmin et al., 2015; Hossain et al., 2016). Considering the earlier findings, the current investigation indicated that high temperature prompted metabolic stress in common carp, creating noteworthy increase in plasma glucose concentration.

In the present study, the substantial reduction of Hb and RBC content may be due to stress caused by the highest temperature. Changes in Hb and RBC content were observed due to thermal stress caused by high temperature in several fish species, including neotropical fish (Carvalho and Fernandes, 2006) and striped catfish (Shahjahan et al., 2018; Islam M. et al., 2019). These reductions of Hb and RBC content might be due to destruction of hematopoietic system under stress caused by the higher temperature. This is surprisingly supported by the increased numbers of Ebs in the blood cells of fish exposed to higher temperature (Table 2). Hematopoietic system has been disturbed in the crucian carp (*Carassius carassius*) exposed to higher temperature (Sikorska et al., 2018). The higher temperature also altered the morphology (nuclear and cellular) of erythrocytes in the current investigation. Significant increases of the frequencies of ENA can occur due to increased production of lipid peroxidation in the blood cells of fish after exposure to the higher temperature (Walia et al., 2013; Bai et al., 2014; Ghaffar et al., 2015). Similarly, significant increases of the frequencies of ECA might be due to morphological changes in the plasma membrane that cause surface deformation and render the erythrocytes more susceptible to burst when traversing small capillaries. It has been reported that there is a cellular abnormality named echinocytic formed due to disruption of the lipid solubility of membranes of erythrocytes (Walia et al., 2013). Similar morphological alterations of erythrocytes due to high temperature have been reported in striped catfish (Shahjahan et al., 2018; Islam M. et al., 2019) and rohu *Labeo rohita* (Ashaf-Ud-Doulah et al., 2019).

Significant increase of WBC content in the highest temperature (35°C) in the present investigation indicated that fish exhibited stress responses at this temperature. Leukocyte counts have been increased in the crucian carp exposed to the higher temperature (Sikorska et al., 2018) is good agreement with our results. These increase of WBC content might be due to the production of higher antibody (Raphael and Kuttan, 2003), a remedial response during exposure to stressed environments (Begg and Pankhurst, 2004; Beyea et al., 2005). Remarkably, formation of neutrophils and lymphocytes showed opposite directions in the highest temperature. Neutrophils increased but lymphocytes decreased significantly in the blood of fish after exposure to the higher temperature. In boars, similar formation of neutrophils and lymphocytes were observed due to stress (Bilandzic et al., 2006). These opposite directions of formation of neutrophils and lymphocytes (Davis et al., 2008) indicated that the higher temperature 35°C may be stressful for the common carp.

The solubility of ambient oxygen decreases with increasing water temperature (Cech and Brauner, 2011). Therefore, fish not only have to deal with thermal stress but also have to challenge hypoxia at higher temperature (Bevelhimer and Bennett, 2000; Chatterjee et al., 2004; Hedayati and Tarkhani, 2014; Ashaf-Ud-Doulah et al., 2019). Though aerators were used in the current experiment, DO (mg/L) decreased significantly in response to the highest temperature. However, DO level was not hypoxic for the experimental fish in the rearing tanks. Thus, stress was undoubtedly induced by raised temperature not by hypoxia.

## Conclusion

In summary, the blood glucose level increased substantially in the highest temperature. The Hb and RBC content decreased but WBC increased significantly in the highest temperature. The number of Ebs, ENA, and ECA were found to be increased at high temperature. The highest temperature significantly increased the number of neutrophils while decreased the number of lymphocytes. Taken all together, this study established that exposure to high temperature beyond certain limit is stressful to the common carp. This is especially important considering the forecasted changes in water temperature due to climate change globally. However, further research is needed to know the impacts of high temperature on growth and reproduction of this fish species after prolonged exposure.

## Data Availability Statement

The datasets generated for this study are available on request to the corresponding author.

## Ethics Statement

The experimental procedure used in this study was approved by the Animal Welfare and Ethical Committee, Bangladesh Agricultural University.

## Author Contributions

MS designed and supervised the experiment, analyzed the data, and drafted the manuscript. MK and MM conducted research and collected the data. SI and MU collected and analyzed the data. MB and SK edited the manuscript.

## Conflict of Interest

The authors declare that the research was conducted in the absence of any commercial or financial relationships that could be construed as a potential conflict of interest.
